# Application of near-infrared fluorescence imaging in lingual lymph node screening and drainage pattern observation for tongue cancer

**DOI:** 10.3389/fcell.2022.986575

**Published:** 2022-09-27

**Authors:** Yaoping Zhu, Tao Xiao, Yijia He, Xiaowei Hong, Ting Zhou, Mingjie Da, Sumeng Ge, Diya Xie, Zhiyong Wang

**Affiliations:** Department of Oral and Maxillofacial Surgery, Nanjing Stomatological Hospital, Medical School of Nanjing University, Nanjing, China

**Keywords:** lingual lymph node, tongue carcinoma, near-infrared fluorescence, indocyanine green, lymphatic drainage

## Abstract

**Objective:** Lingual lymph node (LLN) metastasis is regarded as an indicator of unfavorable prognosis and a crucial sign of the high degree of primary tumor aggressiveness. However, detecting LLN metastasis is an important but frequently overlooked aspect of diagnosis and surgical treatment planning. The study aims to identify LLNs by intraoperative near-infrared (NIR) fluorescence imaging with indocyanine green absorbed into human serum albumin (ICG: HSA) and describe the presence of lymphatic drainage channels from the floor of the mouth in patients with tongue carcinoma.

**Materials and Methods:** 21 patients diagnosed with cT1-T4 squamous cell carcinoma (SCC) of the tongue margin and scheduled to undergo tumor resection and unilateral neck dissection were enrolled. After exposing the neck, the patients were injected with 0.3 ml of ICG: HSA (500 μM) in three quadrants around the tumor, excluding the mucous membrane of the basal region cavity. Employing a near-infrared fluorescence imaging system, the fluorescence of levels I, II, III, and IV was measured during neck dissection.

**Results:** LLNs were detected in four patients and were identified as metastatic LLNs in all 21 patients. The near-infrared fluorescence imaging system showed the existence of lymphatic drainage channels in the floor of the mouth. In patients receiving peritumoral injection of ICG: HSA, the mean fluorescence intensity (MFI)of metastatic lymph nodes (LNs) (178.4 ± 64.39, mean ± SD) was higher than that in non-metastatic LNs (132.0 ± 76.5, mean ± SD) (*p* < 0.05).

**Conclusion:** NIR fluorescence imaging with ICG: HSA could be used for intraoperative identification of LLNs and assist in the determination of metastatic lymph nodes for tongue carcinoma patients. Additionally, this finding demonstrates the feasibility of near-infrared fluorescence imaging in defining lymphatic drainage channels in the head and neck.

## Introduction

The oral cavity is the most common subregion for head and neck cancer. The most common type is squamous cell carcinoma (SCC), which makes up approximately 90% of the cancers found in the oral cavity. The oral tongue has the highest incidence of SCC involvement (35.3%) (Ferlay et al., 2010; Jemal et al., 2011; Weatherspoon et al., 2015). Cervical LN metastasis is one of the most accurate prognostic markers in patients with oral SCC due to the fact that a single metastatic cervical lymph node lowers 5-year survival by 50% (Woolgar et al., 2013).

The LLNs are defined as in-transit lymph nodes that appear infrequently in lymphatic drainage channels. The surgical classifications of LLNs were divided into four groups, including median LLNs, anterolateral LLNs, posterolateral LLNs, and LLNs positioned near the horn of the hyoid bone (Ananian et al., 2015). LLN metastasis should be considered an indicator of poor prognosis and a signal of the high degree of invasion of the primary tumor (Gvetadze and Ilkaev 2020). However, detecting LLN metastasis is an important but frequently overlooked aspect of diagnosis and surgical treatment planning. Preoperative imaging and cautious data analysis are helpful in the identification of LLN metastasis. In cN + -stage patients, MRI and CT can be used to image LLN metastases (Umeda et al., 2010; Ananian et al., 2015; Yang et al., 2018). LLN mapping is frequently conducted using a radiocolloid in the radioisotope (RI) approach. Furthermore, it should be considered whether the sentinel nodes are near the primary tumor; in these cases, the primary tumor may conceal RI accumulation in the small nodes due to the “shine-through” phenomenon (Mahieu et al., 2020). Nevertheless, the use of radiocolloids is limited by the absence of real-time intraoperative visual data and the risk of radiation exposure. LNs can be targeted with a blue dye in tongue SCC; however, the blue dye’s penetration depth is confined and the surgical region is easily contaminated.

Recently, NIR fluorescence imaging has been employed intraoperatively for the localization of lymph nodes, malignancies, and vital organs (Schaafsma et al., 2011). In 1956, ICG lymphography was authorized for use in humans (Giraudeau et al., 2014). NIR fluorescence imaging utilizing indocyanine green (ICG) is safe for human use and poses no risk of radioactive exposure (Tashiro et al., 2017). Since 2007 (Unno et al., 2007), *in vivo* and real-time fluorescent pictures of the lymphatic system and collateral drainage routes have been obtained using this method (Unno et al., 2008; Unno et al., 2011). It is considered a practical, minimally invasive, and safe tool (Adams et al., 2010).

Our study aimed to use NIR fluorescence imaging with ICG: HSA intraoperatively to identify LLNs and to determine the presence of lymphatic drainage channels from the floor of the mouth in patients with tongue carcinoma.

## Materials and methods

### Patients enrolled in this study

The medical ethics committee of the Institute Affiliated Stomatology Hospital, Medical School of Nanjing University, approved this research (2022NL-41). From September 2021 to March 2022, this research included 21 patients with cT1-T4 SCC of the mobile tongue margin who were scheduled for tumor excision and unilateral neck dissection. Neck dissections included resecting levels I, II, and III, as well as level IV in some cases. The primary tumor was removed according to conventional surgical techniques. Exclusion criteria were recurrent oral tongue SCC; oral mucosal disease; earlier head and neck radiotherapy; earlier history of allergy to ICG and iodine; women who were pregnant, lactating, or trying to become pregnant; severe liver or kidney problems (blood biochemical test results more than two times the upper limit of the normal range); and serious cardiac problems (cardiac function class III or under). A written informed consent was obtained from each patient.

### Indocyanine green adsorption to human serum albumin preparation

A total of 25 mg of ICG (Yichuang Pharmaceutical Co., Ltd., Dandong China) was dissolved in 5 ml of distilled water to obtain a concentration of 5 mg/ml ICG. Then, 7.8 ml ICG was transferred to 50 ml Cealb (20% human serum albumin (HSA) solution) to obtain a final concentration of 500 μM ICG: HSA.

### Intraoperative NIR fluorescence imaging

According to our previous studies (Wang et al., 2019), a NIR fluorescence imaging-guided instrument (REAL-IGS, NuoYuan Medical Devices Co., Ltd., Nanjing, China) integrated with a hand-held NIR fluorescence spectrometer (Maya 2000 Pro, Ocean Optics, Dunedin, FL, United States) was employed to obtain NIR fluorescence images, white images and measure fluorescence intensity. The NIR fluorescence images and white images were acquired using an imaging head attached to a pliable gooseneck arm, which allowed it to be positioned in any surgical field. Color videos were obtained by fitting the fluorescence and white images using Fluorescence Navigation Software. A hand-held NIF spectrometer was employed to measure fluorescence intensities. The measurement distance was set to 5 cm, and the angle was set to be perpendicular to the tissue surface.

### Clinical trial

Following neck exposure, 0.3 ml ICG: HSA (500 μM) was injected into three quadrants around the tumor, excluding mucous membranes in the basilar region cavity. We observed intraoperative fluorescence of levels I, II, III, and IV using the NIR fluorescence imaging system with the shadowless lamp in the operating room turned off. Measurements were taken at 5, 10, 15, 20, 25, 30, 45, and 60 min after injection to monitor the lymphatic outflow from the tongue cancer. The images, including white light, fluorescence, and merged images, were transmitted to a digital video processor to be displayed on a monitor in real time. The first NIR fluorescence hotspot was documented, and the LNs that fluoresced were marked. During neck dissection, we searched for fluorescent lymph nodes along the central and lateral directions of the sublingual area, the larger cornu of the hyoid bone, and the lingual vascular system. Intraoperative attentive palpation was also employed to detect LLNs.

LN dissection was performed in accordance with the preoperative plans. Following surgery, all LNs were separated for regular pathological evaluation, and a handheld NIR fluorescence spectrometer was used to evaluate the fluorescence intensities. All LNs were dispatched to a pathology department for a routine pathological investigation that included hematoxylin and eosin (HE) staining and immunohistochemistry (IHC). To determine whether the lymph nodes were colonized by tumor cells, two senior pathologists examined the pathological slides. They were blinded to fluorescence. We collected statistics on the location, number, fluorescence intensity, and histological status of all LNs.

### Statistical analysis

GraphPad Prism Software (Version 9.1.1, La Jolla, CA) was used to analyze the data and generate graphs. Data for comparing the fluorescence intensity between nonmetastatic LNs and metastatic LNs are displayed as the mean ± SD (range). Differences were compared using a two-sided Student’s t test. The receiver operating characteristic (ROC) curve was employed to determine the sensitivity and specificity of peritumoral injection of ICG: HSA for recognizing metastatic LNs. When *p* < 0.05, differences were considered meaningful.

## Results

### Patient characteristics

The study included 21 patients diagnosed with cT1-T4 SCC of the mobile tongue margin and radiological and clinical stage N0. The characteristics of all 21 patients receiving peritumoral injection of ICG: HSA were shown in Table 1. The middle patient’s age was 54.1 years (range, 32–73). The primary tumor site was the anterior portion of the tongue in six patients, the middle portion in six patients, and the posterior portion in nine patients. All patients underwent unilateral neck dissection. Pathological examination revealed that 14 patients had cervical LN metastasis and four patients had LLN metastasis.

**TABLE 1:** Characteristics of patients who received peritumoral injection of ICG: HSA.

Characteristic (*n* = 21)	Absolute no. (%)
Gender	
Male	11 (52.4)
Female	10 (47.6)
Primary tumor site	
Anterior 1/3 of tongue	6 (28.6)
Middle 1/3 of tongue	6 (28.6)
Posterior 1/3 of tongue	9 (42.8)
Clinical T-stage	
T1	2 (9.5)
T2	14 (66.7)
T3	5 (23.8)
Age(y): average (range)	54.1 (32–73)

All first draining NIR fluorescent LNs were considered SLNs. Of the 21 subjects, SLNs were observed in 11 patients within 5 min after injection, in four patients at 10 and 15 min after injection, and in one patient each at 30 and 45 min after injection. No adverse responses or complications associated with ICG: HSA injections occurred during the study.

### Intraoperative NIR fluorescence imaging of lymphatic drainage pathways

**FIGURE 1:**
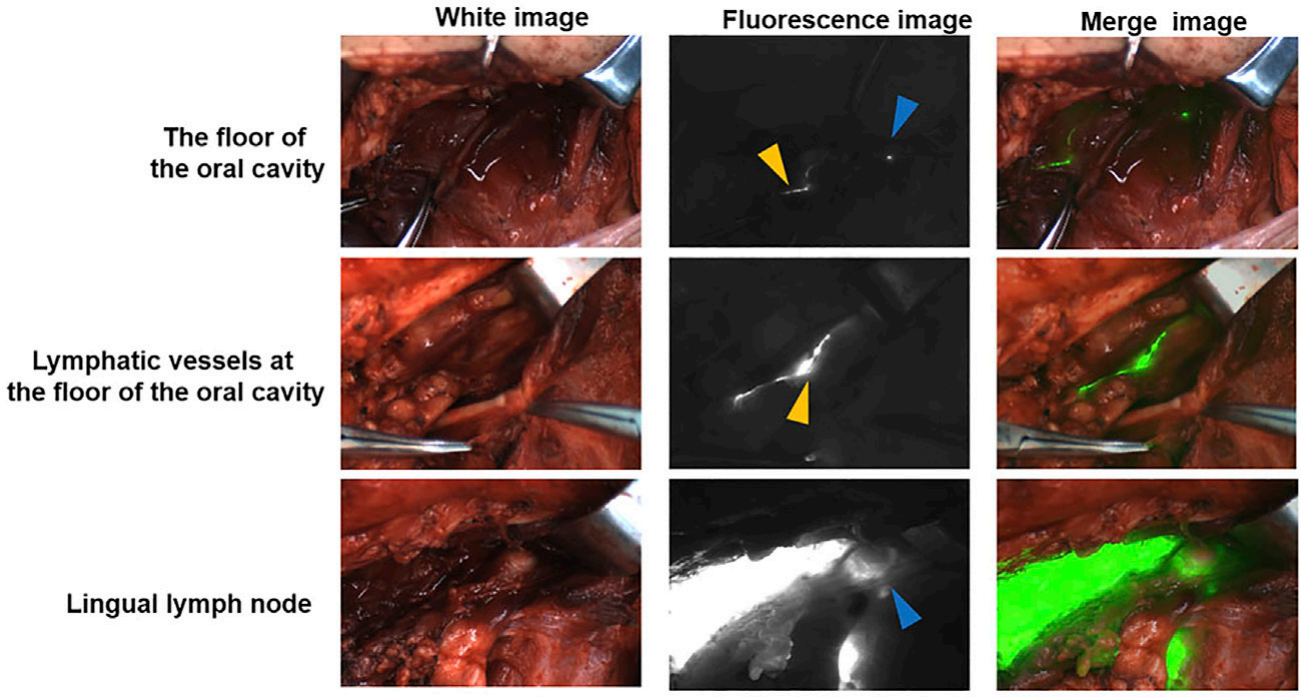
A pathway from the floor of the mouth to the cervical lymph *via* LLN The blue triangle indicates the LLN. The yellow triangle indicates the lymphatic duct.

LLNs were detected in four patients and were identified as metastatic LLNs (Supplementary Figure S1). Among the four patients, LLNs were identified in 3 cases close to the sublingual gland and in 1 case along the greater cornu of the hyoid bone. Trace presence was observed in the lymphatic drainage pathway from the base of the mouth to the neck in patients receiving ICG: HSA peritumoral injections (Figure 1). In that case, a LLN was discovered and confirmed to be a metastatic lymph node. In addition, a lymphatic drainage pathway from the LLN to the sublingual gland was discovered, as well as a descending pathway from level II through the lingual artery. This node was proposed to be located along the lymph drainage path from the tongue margin mucosa to level II.

**FIGURE 2:**
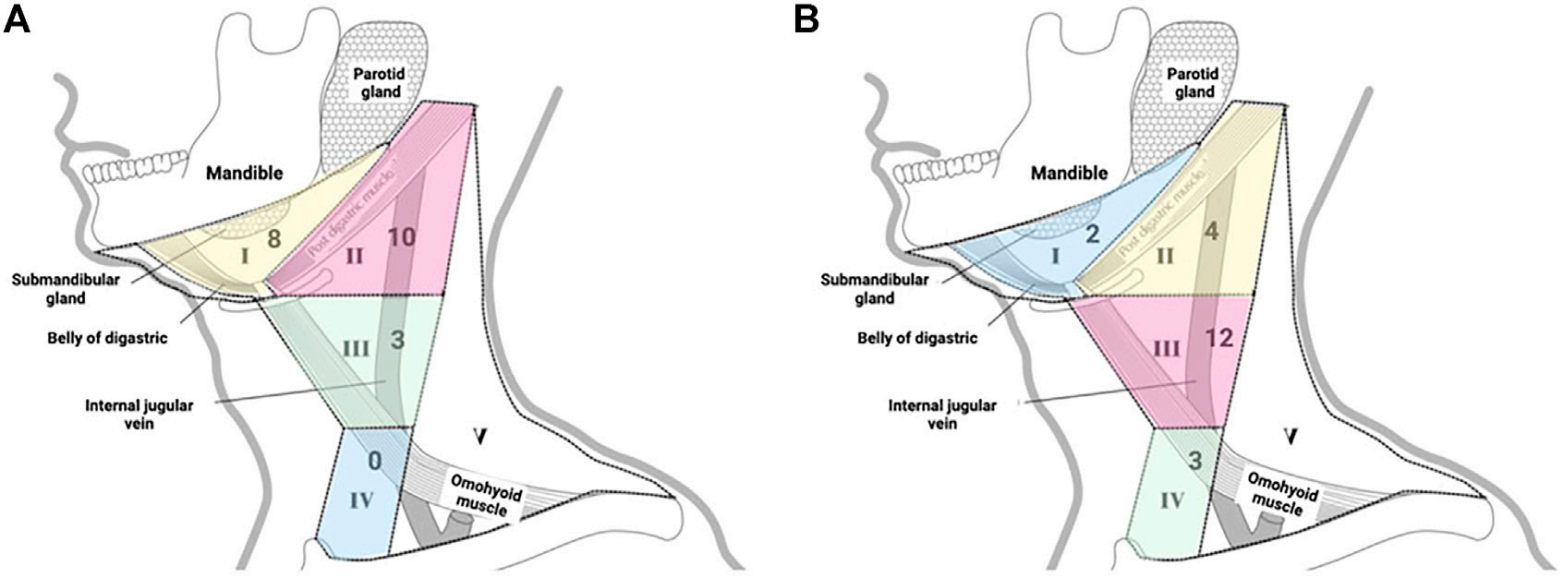
**(A)** Distribution of the first draining NIR fluorescent LN in tongue carcinoma patients; **(B)** Distribution of the metastatic LNs in tongue carcinoma patients.

Due to the different locations of primary lesions, the first draining fluorescent LN and metastatic LNs were found in different regions of the neck. For lesions located in the anterior 1/3 and middle 1/3 of the tongue, the first draining fluorescent LN was most often found in level I (both 4/6). Seven of nine of the first draining fluorescent LNs were found in level II for lesions located in the posterior 1/3 of the tongue (Table 2). For lesions located in the anterior 1/3 of the tongue, metastatic LNs were found in level I and level III. For lesions located in the middle 1/3 of the tongue, metastatic LNs were found in level II and level III. Two-thirds of the metastatic LNs were found in level III for lesions located in the posterior 1/3 of the tongue (Table 3). These results confirm that tongue cancer in different locations has different drainage pathways. However, the first draining lymph node is not necessarily a metastatic lymph node. Except for 4 cases of metastatic LLNs, most of the metastatic LNs were found in level III (12/21, Figure 2).

**TABLE 2:** **(A)** Distribution of the first draining NIR fluorescent LN in tongue carcinoma patients; **(B)** Distribution of the metastatic LNs in tongue carcinoma patients.

Primary tumor site	Anatomical localization of the latero-cervical lymph node levels			
	Ia	Ib	II	III
Anterior 1/3 of tongue	4	0	1	1
Middle 1/3 of tongue	1	3	2	0
Posterior 1/3 of tongue	0	0	7	2

**TABLE 3:** Distribution of the metastatic LNs in tongue carcinoma patients.

Primary tumor site	Anatomical localization of the latero-cervical lymph node levels			
	I	II	III	IV
Anterior 1/3 of tongue	2	0	2	0
Middle 1/3 of tongue	0	3	4	1
Posterior 1/3 of tongue	0	1	6	2

### NIR fluorescence imaging for intraoperative identification of the lymph nodes

**FIGURE 3:**
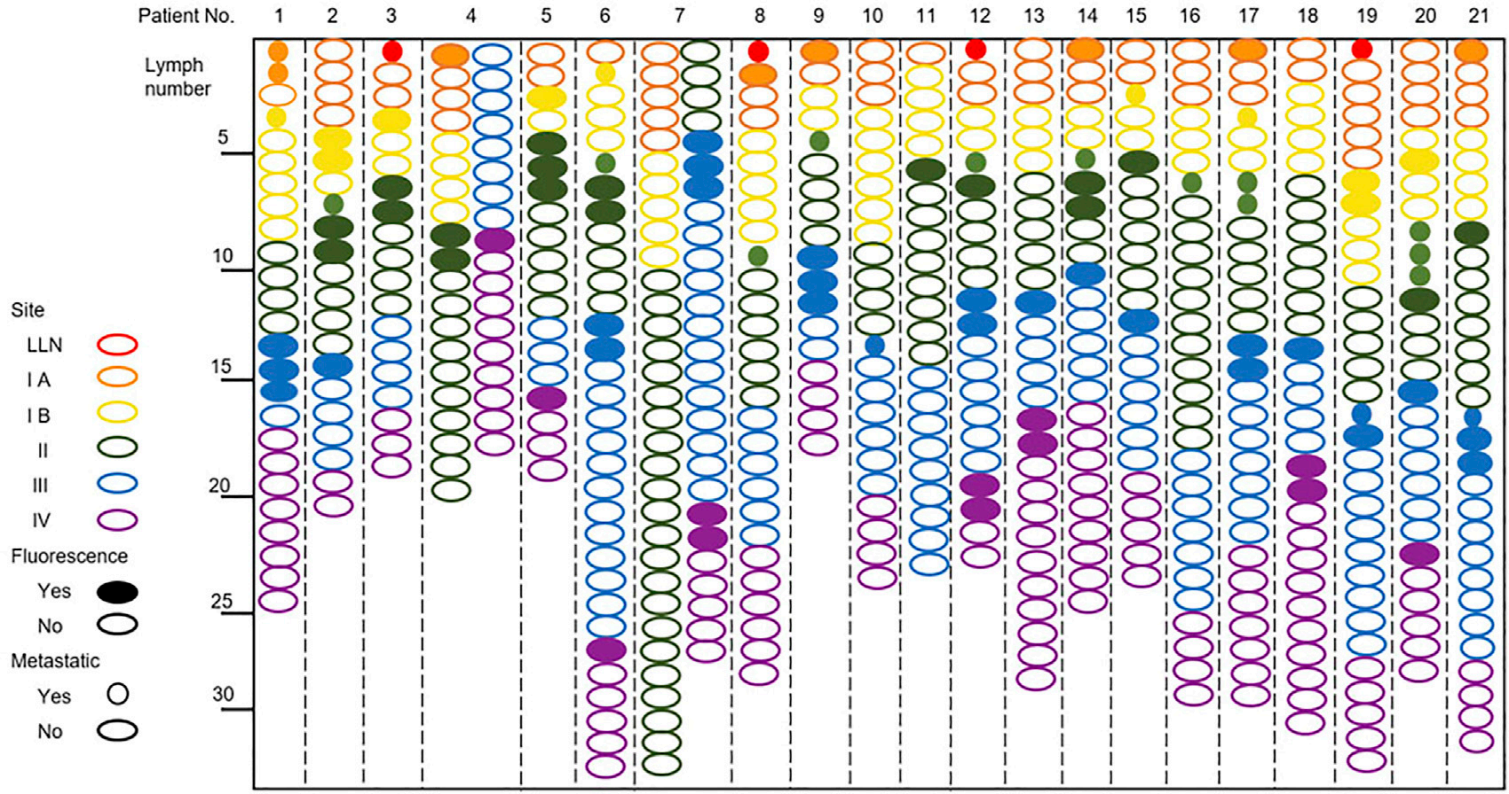
NIR fluorescence imaging for patients with tongue carcinoma receiving a peritumoral injection of ICG: HSA.

**FIGURE 4:**
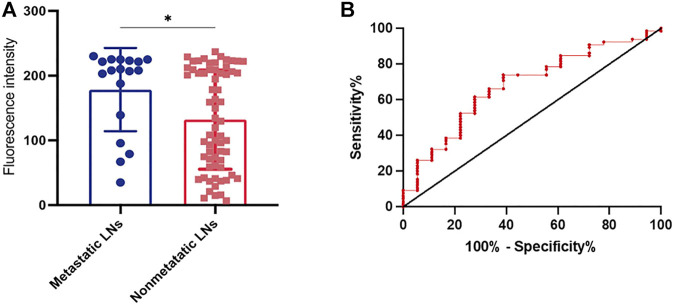
**(A)** The fluorescence intensity of metastatic or non-metastatic LNs after peritumoral injection of ICG: HSA. **(B)** ROC curve analysis of thepatients receiving peritumoral injection of ICG: HSA. ^*^ *P*< 0.05.

There were 92 fluorescence-positive LNs identified in 21 patients; 25 of the 92 fluorescence-positive LNs were recognized as metastatic LNs (Figure 3). Cervical LNs were sorted by region after LN dissection. There were 587 LNs harvested in total, with 66 located in level Ia, 77 located in level Ib, 164 located in level II, 158 located in level III, and 122 located in level IV. The specificity and sensitivity were 88.08 and 100%, respectively; the negative predictive value (NPV) and positive predictive value (PPV) were 100 and 27.17%, respectively; and the accuracy was 88.59% (Table 4). After dissecting and sorting the LNs, we used a handheld NIR fluorescence spectrometer to measure the fluorescence intensities. In patients receiving peritumoral injection of ICG: HSA, the mean fluorescence intensity (MFI) of metastatic LNs (178.4 ± 64.39, mean ± SD) was higher than that in nonmetastatic LNs (132.0 ± 76.5, mean ± SD) (*p* < 0.05) (Figure 4A). The relationship between metastatic status and fluorescence intensity was further analyzed to investigate the possibility of evaluating LN status by measuring fluorescence intensity. According to ROC curve analysis, the AUC of peritumoral injection of ICG: HSA was 0.68 (95% confidence interval (CI) 0.54–0.82) (Figure 4B).

**TABLE 4:** Diagnostic statistics for intraoperative NIR fluorescence imaging.

		Fluorescence-positive	Fluorescence-negative	Total
Pathological examination	Metastatic	25	0	25
	Non-metastatic	67	495	562
	Total	92	495	587

## Discussion

Ozeki et al. (Ozeki et al., 1985) recognized LLNs as potential areas of metastatic spread for oral tongue SCC in 1985. Saito (Saito et al., 2012) and others described a case of T2N0 tongue carcinoma. They discovered a lateral LLN using CT lymphography and a peritumoral injection, raising the probability that LLNs can also act as sentinel lymph nodes. A retrospective study concluded that LLN metastasis (Fang et al., 2019), which is liable to be neglected in tongue SCC patients, is associated with poor survival outcomes. The 5-year locoregional control rate for patients with LLN metastasis was once assumed to be 45%, whereas it was previously predicted to be 65% in sufferers without LLN lesions (Fang et al., 2019). These findings suggest that LLN metastasis is a factor in adverse prognosis. Additionally, it is also an important indicator of high malignancy aggressiveness.

As noted in the literature review, anatomical presence and frequency of metastatic LLN involvement may not be deemed low (Katayama 1943; Mashkov 1968; Ananian et al., 2015; Jia et al., 2018). Medial and lateral LLNs were found in 15.1 and 30.2% of patients, respectively, according to Katayama et al. (Katayama 1943). LLNs were identified in 8.6% of 104 cadavers and classified as regional draining LNs of the oral tongue by Mashkov et al. (Mashkov 1968). Four LLNs were identified out of 21 participants in our study (19.05%), which was in accordance with the research. The LLNs all had metastatic pathology. Moreover, in our present study, we found that LLNs located near the sublingual glands are common, suggesting that we should pay more attention to observing and palpating the sublingual gland area during surgery. The presence of lymph nodes in the floor of the mouth was also confirmed in the current investigation, as illustrated in Figure 1. LLNs may act as intermediate lymphatic components in transit along this drainage channel. Additionally, the findings showed that NIR fluorescence imaging was effective in identifying lymphatic drainage.

As shown in Figure 2, some preferred lymphatic drainage paths were identified: the most common lymphatic drainage pattern was level II of the neck, followed by levels I and III. Our findings appear to contradict the widely held belief that cervical nodal metastases progress in a systematic manner from level I to successive levels (Byers, et al., 1997; Werner et al., 2003). However, the probability of lymph node metastasis in level III of the neck is higher, followed by level II. In particular, some of the first draining NIR fluorescent LNs can be marked as sentinel lymph nodes (SLNs). Another interesting finding is that the area where the metastatic lymph nodes are located is associated with the primary site of the tumor. As illustrated in Table 3, carcinomas in the anterior 1/3 of the tongue tend to metastasize to levels I and III, carcinomas in the middle 1/3 of the tongue tend to metastasize to levels II and III, and carcinomas in the posterior 1/3 of the tongue mostly metastasize to level III. Taken together, our findings indicate that the employment of NIR fluorescence imaging is promising for identifying SLNs inside the cN0 neck in tongue SCC.

Based on our pre-experimental findings, ICG mixed with HSA outperforms ICG alone or methylene blue. As shown in Supplementary Figure S2, there were no lymphatic pathways that could be observed intraoperatively in patients who received peritumoral injection of methylene blue. Moreover, the disadvantage of blue dyes includes limited depth penetration. As illustrated in Supplementary Figure S3, peritumoral injection of ICG alone did not achieve clear NIR fluorescence imaging, which probably resulted from its high dispersion due to its small molecular structure. Therefore, we preferred to use ICG: HSA in this experiment.

To determine the optimal dose of ICG: HSA, we consulted the literature. Previous studies have shown that peritumoral injection with ICG mixed with HSA at a concentration of 500 μM could provide enough contrast to visualize lymphatics, prolong the observation time and achieve more retention in SLNs (Ohnishi et al., 2005; Van der Vorst et al., 2013). Surprisingly, we also identified a link between ICG-HSA fluorescence-positive LNs and metastasis. The sensitivity and specificity of detecting LN status based on NIR fluorescence imaging data were 27.13 and 100%, respectively, with an accuracy of 100%. This demonstrates the viability of employing NIR fluorescence imaging systems with ICG: HSA intraoperatively for fluorescence-guided metastatic LN mapping in patients with tongue carcinoma.

In a study conducted by Bredell et al. (Bredell 2010), the time between injection of ICG (without HSA) and imaging was between 5 and 30 min. We improved the experimental method by premixing ICG with HSA so that most SLNs could be observed within 5 min, and the observation time was relatively broad (5–45 min). Additionally, our findings reported here shed new light on the application of NIR fluorescence imaging with ICG: HSA to intraoperatively identify LLNs and observe the visual lymphatic drainage patterns of tongue carcinoma patients. The insights gained from this study may be of assistance in fluorescence-guided metastasis SLN mapping with NIR fluorescence imaging with ICG: HSA. As a result, if a well-established fluorescence imaging-based SLN mapping technique is used properly, fluorescence-guided surgery can be performed. However, the limitations of the research must be acknowledged. The current study was based on a small sample of participants, which may have lowered statistical power. Therefore, the correlation between ICG-HSA fluorescence-positive LNs and metastasis needs to be assessed in larger patient series.

## Conclusion

Our study concluded that the use of near-infrared fluorescence imaging combined with ICG: HSA for intraoperative LLN detection in patients with tongue cancer is effective. In addition, we confirmed the presence of lymphatic drainage channels in the floor of the mouth, which has important implications for delineating lymphatic drainage channels in the head and neck. Resection of connective tissue on the floor of the mouth at the same time as tongue lesions in patients with tongue SCC is necessary to improve local control. Furthermore, further clinical research on the diagnosis and management of LLN metastases is required before these nodes may be included in the TNM staging scheme. Future investigations should concentrate on optimal dosage, lymphatic tracer enhancement, multimodal hybrid tracers, and surgical procedures.

## Data Availability

The original contributions presented in the study are included in the article/Supplementary Material, further inquiries can be directed to the corresponding authors.
