# Supplementary material for: Application of near-infrared fluorescence imaging in lingual lymph node screening and drainage pattern observation for tongue cancer
Source: Front Cell Dev Biol. 2022 Sep 27;10:986575. doi: 10.3389/fcell.2022.986575 (PMC9552325; doi:10.3389/fcell.2022.986575)

Supplementary Material

**Pathological investigation**

After surgery, all LNs were dispatched to a pathology department for a routine pathological investigation that included hematoxylin and eosin (HE) staining and immunohistochemistry staining of 1000 μm thick paraffin sections (IHC). (Figure S1)

**Peritumoral injection of methylene blue**

Following subplatysmal flap elevation and a total of 1 ml of blue dye, 0.2 ml was injected in the four quadrants surrounding the tumor and at the base and monitored at 5, 10, 15, 20, 25, 30, 45, and 60 minutes. There were no adverse reactions or complications related to the methylene blue injection that occurred during the study period. All of the initial blue-stained lymph nodes were counted. Blue-stained LNs were marked out. Neck resection includes removal of levels I, II, III, and, in some cases, IV. The primary tumor was removed using normal surgical techniques. Resection of the primary tumor was performed according to standard procedures.

**Peritumoral injection of ICG alone**

Following subplatysmal flap elevation and a total of 0.4 ml of ICG alone, 0.1 ml was injected in the four quadrants surrounding the tumor and monitored at 5, 10, 15, 20, 25, 30, 45, and 60 minutes. There were no adverse reactions or complications related to the ICG injection that occurred during the study period. Measurements were taken at 5, 10, 15, 20, 25, 30, 45, and 60 minutes after injection to monitor the lymphatic outflow of tongue cancer. All of the first draining NIR fluorescent hotspots were documented. The LNs that fluoresced were marked. The neck dissections included resectioning levels I, II, and III and, in some cases, level IV. The resection of the primary tumor was performed according to standard procedures.

**Supplementary** Figure **1** Pathological images (HE staining and CK5/6 immunohistochemical staining) of metastatic LLNs and non-metastatic LNs. LLNs, lingual lymph nodes; HE, hematoxylin and eosin.

**
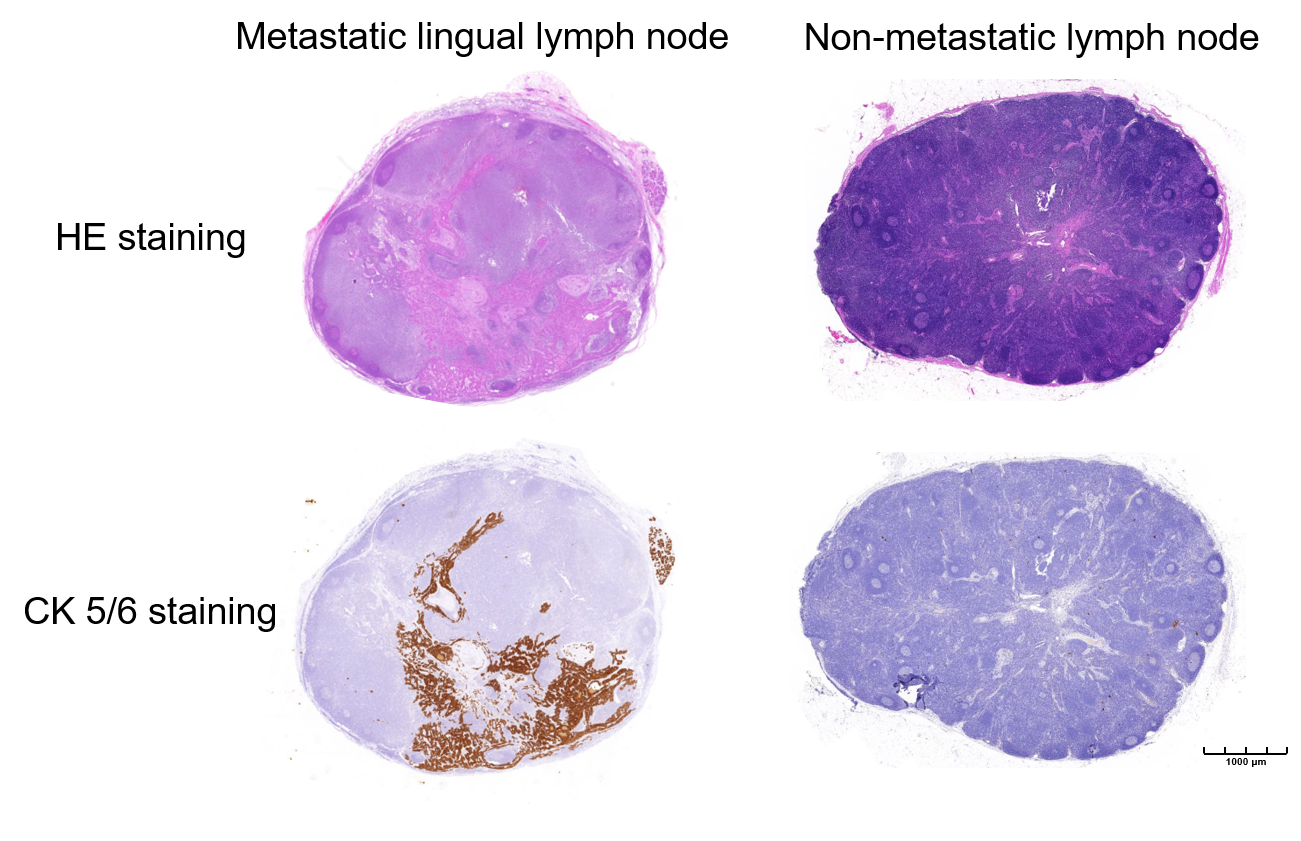
**

**Supplementary** Figure **2** **(A)** 5 min postinjection of methylene blue **(B)** 15 min postinjection of methylene blue **(C)** 25 min postinjection of methylene blue **(D)** 35 min postinjection of methylene blue


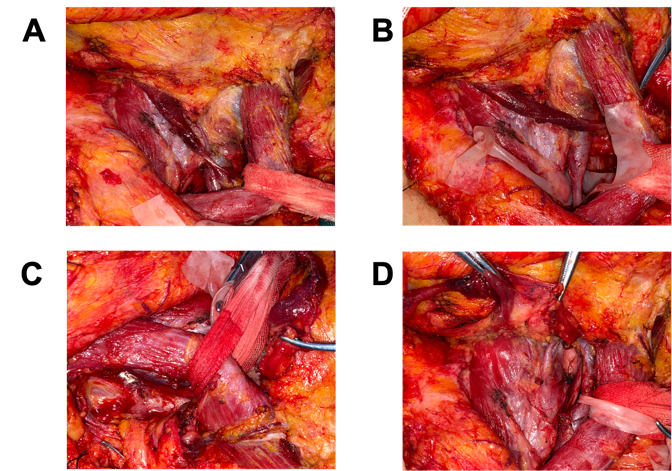


**Supplementary** Figure **3** NIR fluorescence images for patients with tongue carcinoma who received peritumoral injection of ICG alone


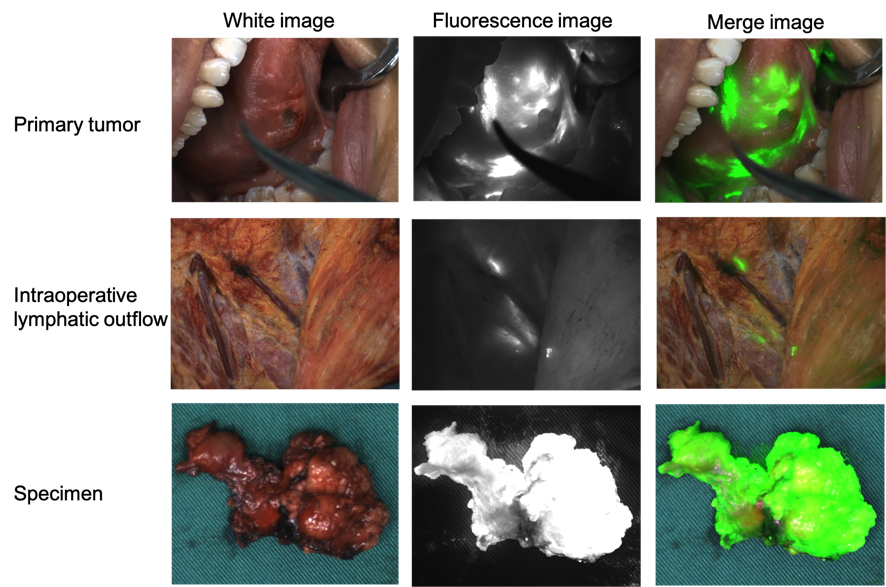

Supplement: Supplementary file 1 [file DataSheet1.docx]
